# Nutritional status, biological maturation and cardiorespiratory fitness in Azorean youth aged 11–15 years

**DOI:** 10.1186/1471-2458-13-495

**Published:** 2013-05-22

**Authors:** Manuel J Coelho-e-Silva, Enio R Vaz Ronque, Edilson S Cyrino, Rômulo A Fernandes, João Valente-dos-Santos, Aristides Machado-Rodrigues, Raul Martins, António J Figueiredo, Rute Santos, Robert M Malina

**Affiliations:** 1 University of Coimbra, Coimbra, Portugal; 2 UEL, Londrina State University, Londrina, Brazil; 3 Department of Physical Education, UNESP – Univ Estadual Paulista (Sao Paulo State University), Sao Paulo, Brazil; 4 Maia Institute of Higher Education (CIDAF), Maia, Portugal; 5 Research Centre in Physical Activity, Health and Leisure, Faculty of Sport, University of Porto, Porto, Portugal; 6 Professor Emeritus, Department of Kinesiology, University of Texas at Austin, Austin, USA; 7 Research Professor, Department of Health and Physical Education, Tarleton State University, Stephenville, Texas, USA; 8 Faculdade de Ciências do Desporto e Educação Física da Universidade de Coimbra, Estádio Universitário de Coimbra, Coimbra, 3040-156, Portugal

## Abstract

**Background:**

Sex and individual differences in biological maturity status can influence height, weight, and body fat. Thus, the rigorous control of these variables seems necessary for estimating overweight and obesity in adolescents. The aims of this study were to estimate the prevalence of overweight and obesity and over-fatness in Azorean adolescents and to examine the contributions of chronological age, sex, estimated maturity status, and cardiorespiratory fitness (CRF) to the risk of overweight and obesity and over-fatness.

**Methods:**

The sample comprised 1,206 youth aged 11–15 years (626 boys and 580 girls) from the Azores Islands, Portugal. Body mass, stature, and skinfolds (triceps and subscapular) were measured. Body mass index (BMI) was calculated and percent fat was predicted from skinfolds. Age- and sex-specific IOTF cut-off values of the BMI defined nutritional status. Biological maturation was estimated as present height expressed as a percentage of predicted adult (mature) stature. The CRF was analyzed from the 20-m shuttle run test.

**Results:**

The total prevalence rates of overweight/obesity and over-fatness were of 31% and 27%, respectively. Low CRF (unfit) and being average and advanced in maturity status were positively and significantly associated with overweight/obesity and with risk of being over-fatness in both sexes.

**Conclusions:**

High prevalence rates of overweight/obesity and over-fatness were identified in Azorean youth, and low CRF and advanced biological maturation were positively associated with overweight/obesity and over-fatness in our sample of adolescents.

## Background

The increased prevalence of overweight and obesity in the pediatric-aged population in several European countries [1] has been consistent with the worldwide epidemic [2]. Overweight and obese children and adolescents are at an increased risk of developing metabolic complications and cardiovascular disease risk factors [3,4].

In Portugal, the combined prevalence of overweight and obesity in Portuguese children 7–9 years, resident on the mainland in 2002–2003, was 29% in boys and 34% girls [5], while corresponding estimates for Portuguese children 6–10 years resident in the Azores (Portuguese Atlantic Islands) in 2001–2002 were 30% in boys and 36% in girls [6]. The latter study also noted higher levels of physical activity (PA) in boys compared to girls and a relationship between PA (very active versus less active) and the risk of obesity [7]. More recently, the combined prevalence of overweight and obesity in a representative sample of Portuguese youth 10–18 years of age in 2011 was 22% in girls and 23% in boys [7].

The evidence from recent surveys of Portuguese youth [5-7] thus suggested that the prevalence of overweight and obesity was higher in girls than boys during childhood (<10 years), whereas the sex difference in prevalence was negligible among adolescents. The indicator of nutritional status was the body mass index (BMI). Unfortunately, the data for adolescents did not ordinarily control for sex and individual differences in biological maturity status which influenced both height and weight. Among youth of the same chronological age, girls tend to be advanced in maturation compared to boys [8].

The literature often assessed maturity status using secondary sex characteristics, clinically- and/or self-assessed [8]. The logistics of assessing secondary sex characteristics is problematic in some settings as they are regarded as invasive. Although valuable clinically, stages of puberty have limitations analytically. Stages of secondary sex characteristics are discrete categories that indicate stage of puberty at time of observation, but provide no information on age of entry into a stage or how long the individual has been in the stage. Moreover, youths at the same stage of puberty who differed in chronological age varied considerably in body size and indicators of fitness [8].

An alternative non-invasive maturity indicator is percentage of predicted mature (adult) height attained at a given age [8]. Mature height is possible to be predicted with a reasonable degree of accuracy from decimal age, height, and weight of the youngster and the heights of their biological parents [9]. The youngster closer to adult height is more mature (advanced in maturation) than the one further removed from adult height. The protocol has been successfully used in studies of PA and youth sports [10-13].

Cardiorespiratory fitness (CRF) was also related to the BMI and maturity status in youths [14-16]. Among Portuguese children 8–10 years of age, an elevated BMI was associated with lower CRF in girls but not in boys [15]. Results of multiple regression analyses among Portuguese youth 8–16 years of age suggested that fatness was inversely and significantly related to CRF, whereas sexual maturity status (self-assessed stages of breast development in girls and pubic hair in boys) accounted for only a small portion of the variance in CRF [16]. Before the obesity epidemic, fatness was equally correlated with fat and fat-free mass [17], but more recent studies suggested that fatness was more correlated to BMI in overweight and obese children [18].

The aims of this study were (1) to estimate the prevalence rates of overweight/obesity and over-fatness and (2) to examine the contributions of chronological age, sex, estimated maturity status and CRF to the risk of overweight/obesity and over-fatness in Azorean adolescents 11–15 years of both sexes.

## Methods

### Setting

The study emerged from a protocol between the Government of the Azores and the University of Coimbra. The Azorean Archipelago consists of nine islands divided into three distinct groups (West, Central and East) located in the North Atlantic between 36° and 43° N and 25° and 31° W. According to 2011 census, the archipelago comprises 240,024 inhabitants [19]. In the 2007–2008 academic year, the educational system included 36 schools and 36,196 students distributed as follows: primary (grades 1–4), 11,928; early elementary (grades 5–6), 6,609; advanced elementary (grades 7–9), 10,231; and secondary (grades 10–12), 7,428. The present study was conducted in 2008–2009, and was part of the third edition of the Azorean Growth Study of secular change in body size, maturation and physical fitness in the school population 10–16 years of age (1988, 1998, and 2008–2009).

The project was approved by the *Committee* of the *University of Coimbra* and registered in the *Portuguese Commission for Protection of Personal Data* [Process #3132006]. Subsequently, the *Azorean Government* issued a written statement to all schools informing them of the objectives of the study. The *Pedagogic Boards* of each school approved the study as a part of the Physical Education program.

### Sample

After obtaining all institutional permissions, participants were selected with a proportionate stratified random sampling strategy taking into account the group of islands (West: Flores, Corvo; Central: Terceira, Faial, Pico, São Jorge, Graciosa; East: São Miguel, Santa Maria). In the West, Central and East groups, 50% of the schools were visited. In Portugal, for each school classes are numbered within each grade and students are numbered within each class. In each chosen school, researchers only contacted PE teachers of even classes and from the selected classes consent terms were uniquely distributed among students having odd numbers. About 9% of the terms were not affirmative. From the same classes, equal portion of students having even numbers were contacted for taking part of the measurements. A total of 1,969 students were examined. The sample for this study was limited to the age range approximating adolescence in girls and boys aged 11–15 years (n = 1,206). Sixteen potential participants did not participate due to health limitations (asthma and flu).

Measurements were obtained by 14 local qualified teachers of Physical Education between November 2008 and March 2009. These observers were master students at the *University of Coimbra* and received one-year training in the protocol and procedures of the study: All signed a contract with the *Government of the Azores* that permitted their participation in research project as part of the master program. Before data collection, observers were trained in anthropometry (27 hours), fitness measurements (27 hours), data management and analysis (27 yours) taught by the first and last authors of the current manuscript.

### Anthropometry

Stature was measured to the nearest 0.1 cm using a stadiometer (Holtain Ltd., Crymmych, Pembrokeshire, UK). Body mass was measured to the nearest 0.1 kg with a portable electronic scale (Tanita Inner Scan BC 532). Subjects wore light indoor clothing with shoes and accessories removed. Subjects were classified by BMI as non-overweight, overweight or obese using the age and sex-specific cut-off values of the International Obesity Task Force (IOTF) [20].

Skinfold thickness was measured to the nearest 0.1 mm on the right side of the body in duplicate with a Lange caliper (Cambridge Scientific Industries, Inc., Cambridge, MD, USA), at the triceps and subscapular. In our laboratory, the intra-observer technical errors of measurement for skinfold thickness in this study were < 5%, measured in duplicate. Percentage body fat was estimated from the two skinfolds [21] and classification of normal-fat and over-fat was done using the cut-off points adopted by the FITNESSGRAM battery [17,22,23]. The prediction equation to estimate percentage of fat for males requires stage of puberty [21]. Consequently stages of pubic hair [24] were self-assessed.

### Predicted mature stature

The Khamis-Roche method [9] was used to estimate mature stature from decimal age, stature and body mass of the participant and midparent stature (average stature of biological parents). The protocol was developed on children from the Fels Longitudinal Study conducted in South-central Ohio in the United States. The median error bound (median absolute deviation) between actual and predicted mature stature at 18 years of age is 2.2 cm in males and 1.7 cm in females [9]. Parent statures were extracted from national identification cards which included stature measured to the nearest centimeter. Measurements were taken by experienced, but not necessarily trained observers. A similar protocol was used in surveys of conscripts in Portugal [25].

Current stature of the youngster was expressed as a percentage of predicted mature stature attained at the time of measurement. This was used as the indicator of biological maturity status. The percentage of predicted mature stature was then expressed as a *z*-score using half-yearly age- and sex-specific means and standard deviations for the longitudinal sample of the *Berkeley Guidance Study*[26]. The rationale for using the Berkeley data has been previously discussed [27,28]. Participants were divided into three maturity groups based on z-scores: Late: z-score less than −1.0; On-time (average): z-score between −1.0 and +1.0; Early: z-score greater than +1.0.

### Cardiorespiratory fitness (CRF)

The CRF was analyzed from the 20-m shuttle run test. This measurement of aerobic performance demonstrated a high correlation with direct assessment of maximal oxygen uptake [29-31]. The 20-m shuttle run test was conducted as described by Léger et al. (29) on an indoor court with slip resistant floor in a 20-m space bounded by two parallel lines. Participants were instructed to run in a straight line, to pivot and turn on completing a shuttle, and to pace themselves in accordance with the audio signals. The initial speed was set at 8.5 km/h (2.4 m/s), which was increased by 0.5 km/h (0.1 m/s) each minute (one minute equals one stage). The CD used was calibrated for duration of one minute. Subjects were advised on proper pacing strategy and motivated to give their best effort. During the test, the subjects were encouraged and verbally communicated at each change of stage. The test ended when the participant stopped due to fatigue, or when they failed to reach the end lines concurrent with the audio signals on two consecutive occasions. The participants were familiar with the test in their physical education classes. Furthermore, the subjects were instructed to refrain from strenuous exercise in the 48 h before testing. All tests were conducted by the same investigators. Based on the 20-m shuttle run scores, participants were classified as fit or unfit relative to age- and sex-specific cut-offs of the FITNESSGRAM [23].

### Analysis

Decimal age was calculated as the difference between date of birth and date of data collection. Descriptive statistics for percentage of predicted mature height, stature, body mass, BMI, percentage of body fat and CRF were calculated and tested for sex differences using analysis of variance (ANOVA) for single year age groups from 11 [11.0-11.9] to 15 [15.0-15.9] years. Preliminary associations between all variables were verified with a matrix of correlation. The associations of weight status and fat status with the independent variables (five age groups, estimated maturity status [late, on time, early], CRF [fit, unfit]) were assessed using chi-square with a Yates correction in 2×2 contingency tables. All independent variables with p ≤ 0.20 in the chi-square test were then inserted simultaneously in multivariate models stratified by sex. The multivariate models were elaborated using the Poisson regression with robust variance (expressed as prevalence ratio [PR] and 95% confidence intervals [95% CI] adjusted for independent variables). In cross-sectional studies with outcomes of higher frequency (>10%), Poisson regression with robust variance is an alternative to logistic regression, which tended to overestimate the magnitude of associations [32]. Poisson regression corresponds to a more conservative multivariate model. The combined effect of weight status (normal and over BMI) and adiposity status (normal and over fat) was tested by comparing the means of the derived four groups using analysis of co-variance (controlling for chronological age). SPSS for Windows (version 17.0) and Stata 8.0 version were used for the analysis. Significance was set at 0.05.

## Results

Means and standard deviations by age group and sex are summarized in Table 1. Body mass did not differ between boys and girls 11 to 14 years of age but boys were significantly heavier at 15 years [p < 0.01]. Girls had a significantly higher percentage body fat from 13 to 15 of age [p < 0.05 to p < 0.01]. Stature did not differ between boys and girls at 11 years of age. Girls were significantly taller at 12 years of age [p < 0.05], and boys were taller at 13–15 years of age [p < 0.01, p < 0.001]. The BMI did not differ between boys and girls 11–13 years of age and at 15 years, but was significantly greater in girls at 14 years of age [p < 0.05]. In contrast to body size and composition, girls were significantly advanced in biological maturation as shown in higher attained percentages of mature stature at each age from 11 to 15 years [p < 0.01]. The number of laps performed in the 20 m shuttle run test was, on average, significantly greater in boys than in girls at all ages from 11 to 15 years of age [p < 0.001].

**Table 1:** Physical characteristics of the subjects by sex and age (n = 1,206)

**Age, years**	**Variables**	**Boys**	**Girls**	***F***	***p***	**ES-r**
		**Mean ± SD**	**Mean ± SD**			
11.0-11.9 yrs	% Predicted mature stature	83.4 ± 3.6	90.7 ± 2.7	229.567	0.000	0.755
Males, n = 92	Body mass, kg	41.6 ± 9.3	41.7 ± 7.9	0.015	0.902	0.000
Females, n = 83	Stature, cm	146.3 ± 6.5	147.9 ± 6.7	2.656	0.105	0.122
	BMI, kg/m^2^	19.3 ± 3.4	19.0 ± 2.9	0.374	0.541	0.045
	Body fat, %	24.0 ± 10.0	23.2 ± 7.4	0.346	0.557	0.045
	20-m shuttle run test, laps	31.3 ± 13.9	22.6 ± 8.6	23.479	0.000	0.335
12.0–12.9 yrs	% Predicted mature stature	87.0 ± 2.5	95.0 ± 3.0	521.255	0.000	0.823
Males, n = 129	Body mass, kg	48.5 ± 12.0	50.7 ± 11.6	2.152	0.144	0.095
Females, n = 121	Stature, cm	152.5 ± 8.3	154.8 ± 7.0	5.989	0.015	0.155
	BMI, kg/m^2^	20.6 ± 4.0	21.0 ± 4.0	0.524	0.470	0.045
	Body fat, %	24.0 ± 11.8	24.9 ± 8.6	0.461	0.498	0.045
	20-m shuttle run test, laps	33.6 ± 16.5	22.1 ± 9.8	43.486	0.000	0.386
13.0-13.9 yrs	% Predicted mature stature	90.9 ± 2.9	97.0 ± 1.6	461.777	0.000	0.787
Males, n = 143	Body mass, kg	53.9 ± 13.0	52.4 ± 9.3	1.382	0.241	0.071
Females, n = 143	Stature, cm	160.1 ± 8.7	157.3 ± 5.7	10.488	0.001	0.190
	BMI, kg/m^2^	20.8 ± 3.8	21.1 ± 3.5	0.415	0.520	0.032
	Body fat, %	21.1 ± 9.1	24.0 ± 6.9	8.844	0.003	0.173
	20-m shuttle run test, laps	36.3 ± 17.3	25.0 ± 12.8	39.230	0.000	0.348
14.0-14.9 yrs	% Predicted mature stature	94.2 ± 2.6	98.7 ± 1.0	297.312	0.000	0.745
Males, n = 120	Body mass, kg	57.9 ± 12.8	56.3 ± 9.6	1.222	0.270	0.071
Females, n = 120	Stature, cm	165.0 ± 8.2	159.2 ± 5.1	43.564	0.000	0.394
	BMI, kg/m^2^	21.1 ± 3.7	22.2 ± 3.8	5.176	0.024	0.145
	Body fat, %	19.9 ± 8.1	27.5 ± 8.9	46.756	0.000	0.405
	20-m shuttle run test, laps	41.0 ± 20.1	21.8 ± 9.0	89.491	0.000	0.522
15.0-15.9 yrs	% Predicted mature stature	97.2 ± 1.7	99.4 ± 0.6	161.914	0.000	0.624
Males, n = 142	Body mass, kg	63.4 ± 13.8	59.1 ± 11.7	7.011	0.009	0.164
Females, n = 113	Stature, cm	170.1 ± 7.7	161.6 ± 5.8	95.636	0.000	0.523
	BMI, kg/m^2^	21.8 ± 4.2	22.6 ± 4.1	2.154	0.143	0.089
	Body fat, %	20.0 ± 9.2	27.5 ± 8.7	44.278	0.000	0.386
	20-m shuttle run test, laps	40.9 ± 19.8	23.2 ± 10.3	74.732	0.000	0.477

*SD* Standard Deviation, *yrs* years, *BMI* Body Mass Index, *ES-r* Effect size correlation.

Prevalence of overweight, obesity, over-fatness and low fitness (unfit) was summarized in Table 2. Boys had a significantly higher prevalence of overweight than girls, while the prevalence of obesity did not differ between the sexes. Data suggested boys as less likely to be classified as normal weight (67%) compared to girls (72%) [p = 0.040]. The prevalence of being over-fat was higher in boys (28%) than in girls (24%) but the difference was not significant [p = 0.154]. An identical trend was noted for the prevalence of being unfit, i.e., higher in boys (56%) than girls (51%), but again the difference was not significant [p = 0.089].

**Table 2:** Prevalence of overweight/obesity, over-fat and cardiorespiratory unfitness by sex and for the total sample

		**Males**	**Females**	**Total**
11 years	N	92	83	175
	Combined prevalence (OW + OB)	37.0%	20.5%	29.1%
	Over-fat	38.0%	22.9%	30.9%
	Unfit	21.7%	16.9%	19.4%
12 years	N	129	121	250
	Combined prevalence (OW + OB)	37.2%	30.6%	34.0%
	Over-fat	36.4%	23.1%	30.0%
	Unfit	49.6%	57.9%	53.6%
13 years	N	143	143	286
	Combined prevalence (OW + OB)	31.5%	24.5%	28.0%
	Over-fat	25.9%	18.2%	22.0%
	Unfit	63.6%	51.0%	57.3%
14 years	N	120	120	240
	Combined prevalence (OW + OB)	30.8%	31.7%	31.3%
	Over-fat	21.7%	28.3%	25.0%
	Unfit	56.7%	65.8%	61.3%
15 years	N	142	113	255
	Combined prevalence (OW + OB)	31.7%	30.1%	31.0%
	Over-fat	22.5%	31.0%	26.3%
	Unfit	74.6%	51.3%	64.3%
Total	N	626	580	1,206
	Combined prevalence (OW + OB)	33.4%	27.8%	30.7%
	Over-fat	28.3%	24.5%	26.5%
	Unfit	55.8%	50.7%	53.3%

The correlation of chronological age with somatic maturation given by attained percentage of estimated mature stature, body size descriptors (stature, body mass, and BMI) and CRF is presented in Table 3. This table also includes the coefficients of partial correlation between body size descriptors, somatic maturation and CRF (controlling for the spurious effect of decimal age).

**Table 3:** Bivariate correlations (coefficient and 95% CI) between chronological age, indicator of somatic maturation, body mass, stature, BMI, estimated percentage of fat mass and 20-m shuttle run performance (upper portion) and partial correlations (coefficient and 95% CI) controlling for chronological age (bottom portion) for the total sample (n = 1,206)

		**% PMS**	**Body mass**	**Stature**	**BMI**	**Body fat**	**20-m SRT**
*Bivariate correlation*	CA	0.71 ** (0.68 to 0.74)	0.48 ** (0.44 to 0.52)	0.64 ** (0.61 to 0.67)	0.23 ** (0.18 to 0.28)	−0.02 (−0.08 to 0.04)	0.13 ** (0.07 to 0.19)
*Partial correlation*	% PMS		0.36 ** (0.31 to 0.41)	0.31 ** (0.26 to 0.36)	0.30 ** (0.25 to 0.35)	0.26 ** (0.21 to 0.31)	−0.30 ** (−0.35 to −0.25)
*(controlling for CA)*	Body mass		—	0.54 ** (0.50 to 0.58)	0.90 ** (0.89 to 0.91)	0.59 ** (0.55 to 0.63)	- 0.19 ** (−0.24 to −0.13)
	Stature			—	0.13 ** (0.07 to 0.19)	−0.01 (−0.07 to 0.05)	0.12 ** (0.06 to 0.18)
	BMI				—	0.72 ** (0.69 to 0.75)	- 0.29 ** (−0.31 to −0.24)
	% body fat					—	−0.35 ** (−0.40 to −0.30)

*CA* Chronological age, % *PMS* Predicted Mature Stature, *BMI* Body Mass Index, *SRT* Shuttle Run Test.

* *p* < 0.05.

** *p* < 0.01.

**Figure 1:**
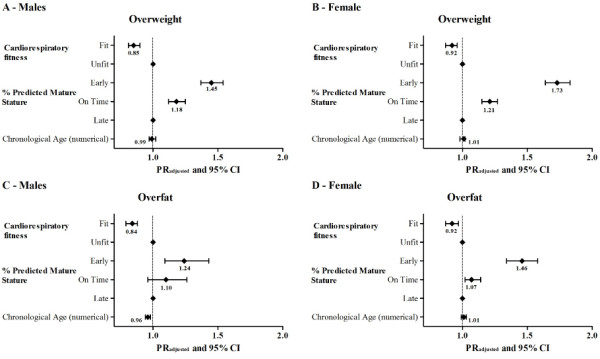
Prevalence ratio (PR) for variables included in the model and confidence intervals (95% CI) for risk of overweight (Males - IA and Females IB) and overfat (Males - IC and Females ID) in relation to age, maturity and level of CRF by sex.

Associations (crude and adjusted) of weight status and fat status with age group, estimated maturity status and CRF status were summarized in Table 4 and illustrated in Figure 1. Chronological age group was not significantly associated with weight status in both sexes and with over-fatness in girls. However, age group was significantly associated with being over-fat in boys [PR = 0.96, 95% CI: 0.94 to 0.99, p < 0.01]. Youths who are on-time (average) and early in maturity status were more likely to be classified as overweight or obese than youths who were late in maturity status [on time: males PR = 1.23, 95% CI: 1.18 to 1.27, females PR = 1.21, 95% CI: 1.15 to 1.27; early: males PR = 1.52, 95% CI: 1.46 to 1.59, females PR = 1.77, 95% CI: 1.67 to 1.86]. For over-fatness, only early maturing boys were more likely to be over-fat [PR = 1.29, 95%CI: 1.01 to 1.52], while on-time [PR = 1.07, 95% CI: 1.01 to 1.13] and early [PR = 1.48, 95% CI: 1.38 to 1.60] maturing girls were more likely to be over-fat.

**Table 4:** Crude prevalence ratio (PR) and confidence intervals (95% CI) for overweight and over-fat relative to chronological age, estimated biological maturity status and cardiorespiratory fitness categories for males (n = 626) and females (n = 580)

			**Poisson regression**	
**Sex**	**Variable**	**Category**	**Overweight and obese**	**Over-fat**
			PR crude	PR crude
Males	Chronological age		0.99 (0.97-1.01)	0.96 (0.94-0.99)
	% Estimated mature stature	Late	1	1
		On time	1.23 (1.18-1.27)	1.11 (0.95-1.30)
		Early	1.52 (1.46-1.59)	1.29 (1.01-1.52)
	Cardiorespiratory fitness	Unfit	1	1
		Fit	0.86 (0.81-0.91)	0.86 (0.82-0.91)
Females	Chronological age		1.01 (0.99-1.03)	1.02 (0.99-1.04)
	% Estimated mature stature	Late	1	1
		On time	1.21 (1.15-1.27)	1.07 (1.01-1.13)
		Early	1.77 (1.67-1.86)	1.48 (1.38-1.60)
	Cardiorespiratory fitness	Unfit	1	1
		Fit	0.87 (0.82-0.91)	0.88 (0.84-0.93)

Overweight and obesity by IOTF criteria [17]; Over-fat, >25% for boys and >30% for girls [19,20]; CRF categories were based on age- and sex-specific cut-offs of the FITNESSGRAM [23].

## Discussion

The prevalence of overweight and obesity was 31% among Azorean youth 11–15 years of age, for both sexes, while the prevalence of over-fatness was 27%. About 64.3% of Azorean adolescents were classified as having a normal weight and normal fat, while 239 youth (19.8%) were classified both over-fat and overweight/obese. The two classifications thus agreed in 84.2% of cases (boys: 85.3%; girls: 82.9%). Among youth discordant for fatness and weight status, 82 youth (6.8%) were over-fat but classified as having a normal weight status by the BMI, while 111 youth (9.2%) were classified as having an acceptable level of fatness but were classified overweight or obese according to the BMI. The current study (like *FITNESSGRAM*) used skinfolds as the field method to estimate body fatness. Skinfolds had proven to be one of the most effective field methods for estimating body fatness with standard errors of estimate of 3 to 4% body fat. Although BMI is often viewed as an alternative to estimates of body fatness, however, it is not as effective in identifying moderately over-fat children [33].

The observed prevalence of overweight and obesity in the present study of Azorean youth, aged 11–15 years, was 33% in males and 28% in females (Table 2). This prevalence was higher than estimates for a larger sample of Portuguese youth, 10–18 years of age, which was 23% for males and 21% for females [7]. Both surveys used the IOTF criteria. Studies differed, of course, in consent rate, sampling, and so on, but the possibility of differences in lifestyle between the islands and the mainland needs consideration. A recent study noted an inverse association between intake of milk intake with BMI and percentage of body fat in Azorean girls [34].

The relationship between variability in chronological age, maturity status and CRF, on one side, and inter-individual variability in the combined prevalence of overweight/obesity and over-fatness, on the other side, was also considered in the present study. Percentage of predicted adult height attained at the time of measurement was used as a non-invasive parameter of biological maturity status. Youth of both sexes classified as on-time (average) and early maturing had a higher probability of being classified as overweight or obese. In contrast, only early maturing boys were more likely to be classified as over-fat, while both on-time and early maturing girls were more likely to be over-fat compared to late maturing boys and girls, respectively. These results were consistent with an earlier study of Portuguese boys and girls 10–15 years of age; early maturation based on clinically-assessed stages of puberty (genitalia for males, breasts for females) was associated with an increased risk of overweight/obesity [35].

However, it is worth highlighting that the mentioned study [35] grouped the sample using the quartiles of the decimal age adjusted for stages of sexual maturation and sex. For example in stage 1, children were considered as ‘early maturers’ if they were in the first quartile of the decimal age for that stage and sex. The same procedure was used for the other stages. Those in the fourth quartile of the decimal age were considered as ‘late maturers’. Afterwards, in the data analysis, differences in the prevalence of overweight among the quartiles of sexual maturation were tested using a Chi-square test, separately for boys and girls. Erroneously, the literature often interprets sexual maturation given by stages of pubic hair interpreted as an indicator of timing.

Current stature expressed as a percentage of the predicted mature value has been used in studies of PA [36,37]. A model for adolescent involvement in PA that incorporates individual differences in timing and tempo of biological maturation has been recently presented in a cross-sectional sample of adolescents [28]. The decline in PA with age was related to physical and physiological changes associated with pubertal maturation and growth spurt, which include changes in body composition and body proportions.

Low CRF has been associated with the presence of cardiovascular and metabolic risk factors in youth [38,39]. Low levels of CRF in childhood and adolescence have also been associated with increased cardiovascular risk in adulthood [40]. About 53% of Azorean youth in the current study were classified as unfit in the 20-m shuttle run test. More importantly, unfit boys and girls were more likely to be overweight/obese than aerobically fit youth. In cross-sectional [16] and longitudinal [41] analysis from Portuguese youth, low levels of CRF were associated with increased BMI and body fatness. Note, however, that biological maturation was positively related to both CRF, especially in boys, and fatness during adolescence [16,42]. These relationships need further studies. Allowing for the limitation of the current study, boys and girls who were simultaneously over-fat and had a higher BMI tended to be more advanced in estimated maturity status and were also less fit aerobically (Table 4). BMI may temporarily be confounded by inter-individual differences in the timing of the growth spurts of height and weight. This, unfortunately, was not captured in cross-sectional surveys. As such, use of a multi-method approach may help to avoid potentially erroneous classification of BMI for chronological age during adolescence. These issues influence the diagnostic accuracy of BMI to diagnose overweight/obesity status among adolescents. The issue has been examined in adults [43] and more recently in youth [18].

Previous studies with Azorean adolescents indicated a significant association between higher levels of PA and a lower occurrence of overweight and obesity, and metabolic risk [6,44]. The results of these studies and also of the present study claim a need for promotion of PA programs in Portuguese youth. Organized sport may be a relevant correlate. Portuguese adolescents spent 11% to 13% of total daily energy expenditure in organized sports, which corresponded to 35% to 42% of the moderate-to-vigorous portion of daily energy expenditure [45]. Unfortunately, many currently organized sport programs do not fit the interests and readiness level of the overweight and obese youngster.

Although the results highlighted an association between low CRF, advanced biological maturation, weight status and over-fat in youth, several limitations of the study should be noted. First, the cross-sectional design of the current study precludes causal inferences to be drawn. Second, PA was not assessed. Third, a non-invasive indicator of maturity status was used. Although the results were consistent with several studies using secondary sex characteristics, validation of attained height expressed as a percentage of predicted adult height is needed.

However, the observed associations between sexual maturation and obesity presented in the current study may have important implications for the classification, management, and prevention of child and adolescent obesity. There is a risk to uncritically apply BMI cutoffs for classifying child and adolescent overweightness and obesity without fully recognizing their limitations and the potential misclassifications. To our knowledge, none of the existing anthropometry references are able to account for maturation status.

## Conclusions

High prevalence rates of overweight/obesity and over-fatness were identified in Azorean youth, and low CRF and advanced biological maturation were positively associated with overweight/obesity and over-fatness in our sample of adolescents.

## Abbreviations

CRF: Cardiorespiratory fitness; BMI: Body mass index; IOTF: International obesity task force; PA: Physical activity; UK: United Kingdom; ANOVA: Analysis of variance; PR: Prevalence ratio; CA: Chronological age; % PMS: Predicted mature stature; BMI: Body mass index; SRT: Shuttle run test.

## Competing interests

The authors declare that they have no competing interests.

## Authors’ contributions

MJCS: (1) conception and design of the study, (2) acquisition, analysis and interpretation of data, (3) draft of the article and selection of manuscripts to discuss the results and (4) final approval of the version to be submitted. AJF: (1) acquisition of data, (2) revising it critically for important intellectual content, (3) final approval of the version to be submitted ERVR, RAF: (1) analysis and interpretation of data, (2) revising it critically for important intellectual content, (3) final approval of the version to be submitted. ESC, JVS, AMR, RM: (1) revising it critically for important intellectual content, (2) final approval of the version to be submitted. RMM and RS: (1) revising it critically for important intellectual content, (2) selection of manuscripts to discuss the results, (3) final approval of the version to be submitted, (4) final editing for corrections in the English quality. All authors read and approved the final manuscript.

## Pre-publication history

The pre-publication history for this paper can be accessed here:

http://www.biomedcentral.com/1471-2458/13/495/prepub
